# Profile of Splash, Sharp and Needle-Stick Injuries Among Healthcare Workers in a Tertiary Care Hospital in Southern India

**DOI:** 10.7759/cureus.42671

**Published:** 2023-07-29

**Authors:** Praisie R, Anandadurai D, Sudhir B Nelson, Sriandaal Venkateshvaran, Manoje Thulasiram

**Affiliations:** 1 General Medicine, Velammal Medical College and Hospital, Madurai, IND; 2 Community Medicine, Velammal Medical College Hospital & Research Institute, Madurai, IND; 3 Community Medicine, Velammal Medical College and Hospital, Madurai, IND

**Keywords:** hiv, hepatitis, exposure, needle stick injury, healthcare workers

## Abstract

Background and objective

Among biological hazards faced by healthcare workers, one of the most commonly reported is sharp-related injury. Needle-stick and sharp injuries transmit infectious diseases, especially blood-borne viruses. The conditions in which these exposures occur vary. Reporting of exposure and proper post-exposure prophylactic measures are important in controlling blood-borne infections in healthcare workers. Better analysis of such accidents is an important tool to reinforce exposure preventive measures. Hence, we conducted a study to describe the profile of splash, sharp and needle-stick injuries among healthcare workers in a tertiary care hospital.

Methods

A record-based cross-sectional study was done in a tertiary care hospital, in south Tamil Nadu. All healthcare workers who have reported needle stick, sharp, or splash injuries during the last five years in the hospital were included. Data was extracted from post-exposure reports maintained in the hospital. Results are expressed in mean, standard deviation and percentages.

Results

A total of 189 healthcare workers (HCWs) had reported instances of splash, sharp and needle stick injuries in the last five years. The most common exposure was needle prick (86.2%), followed by splash of fluids (7.4%). The majority of HCWs were from the nursing department (44.4%), and the most commonly reported place of exposure was the Emergency Department and Intensive Care Unit (ICU) (30.3%), followed by inpatient wards. The associated activity in the majority of the injuries/exposures was the transfer of sharps or cleaning surfaces (26.4%), followed by blood withdrawal (25.7%). After the exposure, 99.5% of HCWs washed the wound immediately. In a total of 135 exposures, the identity of the source, and thus, the serological status was known. Among these, hepatitis B was the most common (17.8%), followed by HIV (11.9%). All exposures related to unknown sources were considered positive exposure and were managed accordingly. Among the HCWs with possible seropositive exposure to hepatitis B, antibody titres were recorded and HBV Immunoglobulin (low titre), and vaccination were administered accordingly. Among the possible HIV exposures, 97.1% of HCWs initiated post-exposure prophylaxis (PEP). All probable hepatitis C exposures were given counseling and advised to follow up. No seroconversion at six months of follow-up has been recorded till now.

Conclusion

Healthcare workers are constantly at risk of exposure to splash, sharp and needle stick injuries, and although all categories of workers are at risk, nurses are at particularly high risk. A variety of activities can result in injury or a splash of fluids and so preventive activities, including health education, should be focused on all areas of healthcare and for all healthcare workers. More awareness is needed among healthcare workers regarding post-exposure prophylaxis.

## Introduction

Medicine is a field where there is a high potential for occupational health problems. Its workers work in close contact with patients, who are a potential source of infection. Occupational hazards among healthcare workers include both biological and non-biological hazards. Among biological hazards, one of the most commonly reported is sharp-related injury. The conditions in which these exposures occur vary, and include manipulation of needles, recapping needles, during any procedure/surgery, collision with another person or object, and during clean-up and disposal [1]. Needle-stick and sharp injuries transmit infectious diseases, especially blood-borne viruses. Concern includes the human immunodeficiency virus (HIV), which leads to AIDS (acquired immune deficiency syndrome), hepatitis B (Hep B), and hepatitis C (Hep C) [2]. Incidental punctures by contaminated needles can inject hazardous fluids into the body through the skin. There is potential for injecting hazardous drugs, but contact with infectious fluids, especially blood, is by far the greatest concern. Even small amounts of infectious fluid can spread certain diseases effectively. Infection can also spread when bodily fluids splash over the skin, eyes, mouth, or other mucosal surfaces. Sharps can create a cut in the skin, allowing contact with blood, or fluids. The risk of infection after exposure to infected blood varies with the blood-borne pathogen [3]. It is reported that approximately 3 million healthcare workers are exposed to blood-borne infections, with hepatitis B being the most common, followed by hepatitis C and HIV [4]. The Indian Council for Medical Research (ICMR) has come out with guidelines for the control of blood-borne infections among healthcare workers. It includes information on continuous health education and training on standard operating procedures to be followed while caring for patients. In addition, it includes clear instructions on what to do following exposure. Reporting of exposure and proper post-exposure measures are important in controlling blood-borne infections in healthcare workers [5]. Various studies in India have shown that factors influencing exposure vary from center to center and there are gaps between knowledge and practice related to HIV prevention. Needle-stick and sharp injuries (NSSIs) continue to pose a serious occupational problem [6, 7].

When a healthcare worker (HCW) is accidentally exposed to a needle stick injury or sharp injury, or a splash of body fluids on mucous membrane or broken skin, they are instructed to wash it immediately and report to the casualty as soon as possible. The casualty medical officer immediately contacts the infection control nurse and post-exposure prophylaxis (PEP) medical officer to report the incident. Tetanus toxoid (TT) is given in the emergency department if indicated. The source is traced by the infection control nurse. If the source is known and the viral marker reports are available during the current admission, no further tests are needed for the source. If viral markers are unavailable, a sample is sent for testing immediately, and the results are recorded. If the source is known and negative for all three, HCWs are reassured, the risk of the window period is explained, and follow-up is advised. If the source is unknown, it is considered to be positive for HIV, Hep B, and Hep C. The healthcare worker is simultaneously tested for viral markers and Hep B titre.

If the source is HIV positive or if the source is unknown, the first dose of post-exposure prophylaxis (PEP) for HIV is given immediately or within 72 hours and is advised to continue for 28 days.

If the source is hepatitis B positive or unknown, and if the anti-HbsAg (Hepatitis B surface antigen) titre > 10 (protective), HCW is reassured and advised to follow up. If anti-HbsAg titer < 10 (HCW is either unvaccinated or incompletely vaccinated, or a non-responder with full vaccination), HCW is advised to take Hep B immunoglobulin, preferably within 12 hours, along with Hep B vaccination simultaneously. The HCW should complete the Hep B vaccination schedule if unvaccinated or incompletely vaccinated.

If the source is Hep C positive, or if the source is unknown, HCW is informed of the risk of transmission of Hep C, the unavailability of a vaccine for Hep C, and the need for follow-up with anti-Hep C after three months. All the incidents are recorded in PEP forms and maintained by the infection control department.

Preventing injuries is the most effective way to protect workers. Hence, we conducted this study to review the epidemiology of splash, sharp and needle-stick injuries among HCWs, and describe the circumstances under which these injuries occurred. By doing so, we aim to contribute to the overall knowledge database, as better analysis of such accidents is an important tool to put in place exposure preventive measures.

## Materials and methods

Study design, setting, and study participants

This is a record-based cross-sectional study done in a 1200-bed tertiary care hospital, in south Tamil Nadu. By using the universal sampling technique, all healthcare workers who have reported needle stick, sharp, or splash injuries during the last five years (2018-2023) in the hospital were our study participants.

Data collection tools and methods

The institutional infection control board maintains all the self-reported questionnaires filled out by the exposed healthcare worker. Data was extracted from these questionnaires and the attached laboratory reports. The questionnaire included information regarding socio-demographic details like age, gender, department, and category of HCW. Part II contains information regarding the type of injury, the source of injury (known/unknown), hepatitis B vaccination status, immediate post-exposure measures taken like washing of hands, the status of the source of exposure, and if the HCW knew his/her status of HIV, HBV, or HCV positivity. Part III has information regarding the screening for HIV, Hep B, and Hep C viruses, and the immediate treatment measures taken.

Statistical methods

Data was entered in MS Excel, and analysis was done using PSPP version 1.6.2 software. Results are expressed in mean, standard deviation, frequency and percentages.

Ethical consideration

Clearance from the institutional ethical committee was obtained before the start of the study (IEC No: VMCIEC/001/2023), and permission from the institution head was obtained to access the records maintained in the teaching hospital.

## Results

A total of 189 healthcare workers (HCW) had reported instances of splash, sharp and needle-stick injuries in the last five years. The mean age of the reported workers was 28.47±8.5 with the minimum age being 18 years and a maximum of 53 years. The majority of the workers were female (84.1%).

**Figure 1:**
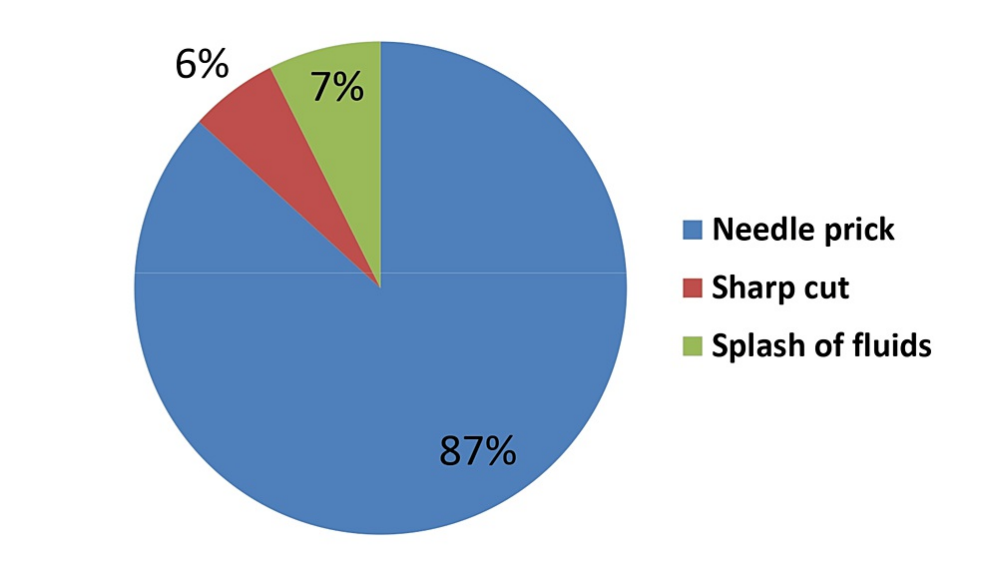
Nature of the exposure

The most common exposure was needle prick (86.2%), followed by a splash of fluids (7.4%), while 5.8% had sharp cuts (Figure 1).

**Figure 2:**
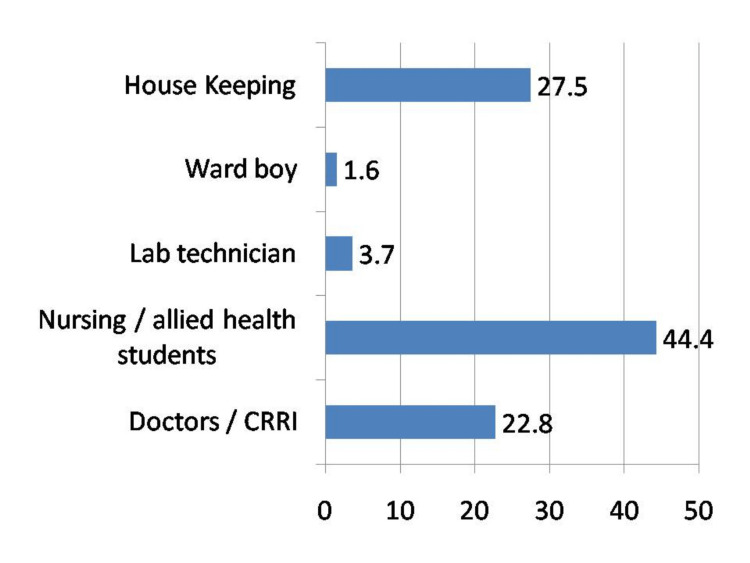
Distribution of healthcare workers who reported exposure according to their job description CRRI: Compulsory Rotatory Residential Internship

Among the healthcare workers who reported exposure to blood and body fluids, 44.4% were from the nursing department, 27.5% were from the housekeeping department, 22.75% were doctors, and less than 5% were ward boys and technicians (Figure 2).

The emergency department and intensive care unit (ICU) together formed the most common location of exposure (30.3%), followed by inpatient wards.

The majority of the exposures, i.e., 26.4%, were during the transfer of sharps or cleaning tables, cots, floors, etc., and this injury was most commonly reported by housekeeping staff. In a rare instance, a housekeeping staff member was exposed to splash while standing nearby as blood was being drawn from a patient. If housekeepers were excluded, then the most common instance of exposure was while drawing blood (25.7%), followed by minor procedures/surgery (21.4%) (Table 1).

**Table 1:** Activities associated with exposure (excluding housekeeping) IV: Intravenous; IM: Intramuscular; SC: Subcutaneous

Procedure	Doctors/Interns	Nursing	Others	Total (% within procedure)
While drawing blood	10 (27.8%)	20 (55.6%)	6 (16.7%)	25.7%
Minor procedure/surgery	22 (73.3%)	8 (26.7%)	0	21.4%
While giving injection (IV/IM/Sc)	4 (19%)	16 (76.2%)	1 (4.8%)	15%
While recapping/disposing of needle	2 (9.1%)	18 (81.8%)	2 (9.1%)	15.7%
IV cannula insertion/removal	5 (23.8%)	16 (76.2%)	0	15%
While transporting waste/cleaning	0	7 (77.8%)	3 (22.2%)	0.7%

Hepatitis B vaccination

Among the HCWs, 29.6% were not aware of their hepatitis B vaccination status. 41.3% had completed their full vaccination schedule, 24.9% were partially immunized and 4.2% were not vaccinated.

Post-exposure care

A total of 99.5% of healthcare workers washed the wound immediately after the exposure, while a small proportion, i.e., 1.6%, did not follow the precaution of washing immediately. Of all the HCWs who were exposed, 82.5% were immunized with TT soon after the exposure. A total of 16% of HCWs were recently immunized, while the rest were not willing to undergo immunization.

Exposure to known positive source

In a total of 135 exposures, the identity of the source, and thus, the serological status was known. Among these, hepatitis B was the most common (17.8%), followed by HIV (11.9%) (Table 2).

**Table 2:** Exposure serology from known source (n = 135)

Serological Status		Frequency	Percentage
Human Immunodeficiency Virus (HIV)	Positive	16	11.9%
	Negative	119	88.1%
Hepatitis B Virus (HBV)	Positive	24	17.8%
	Negative	111	82.2%
Hepatitis C Virus (HCV)	Positive	14	10.4%
	Negative	121	89.6%

All exposures related to unknown sources was considered a positive exposure and was managed accordingly.

Hepatitis B

**Figure 3:**
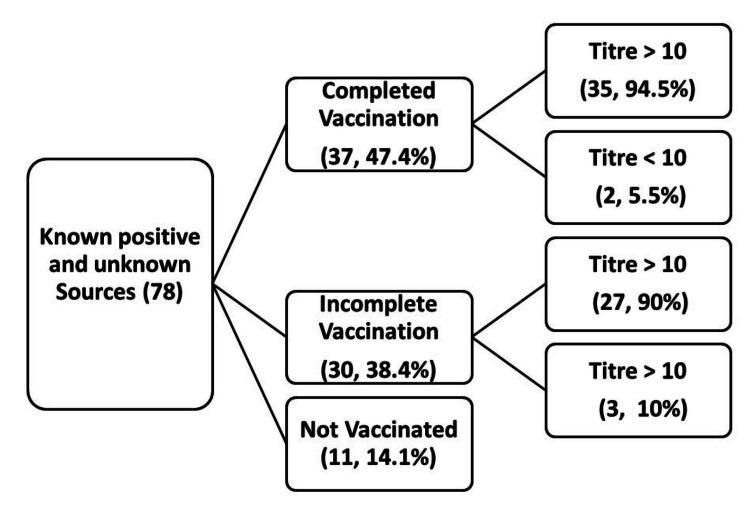
Healthcare workers with possible seropositive exposure to hepatitis B

Among the healthcare workers with possible seropositive exposure to hepatitis B (positive among known sources - 24 + unknown sources - 54 = 78), 47.4% were fully vaccinated healthcare workers (n = 37) (Figure 3).

Among those who completed vaccination, the majority (94.5%) had protective titre and required only reassurance. The rest, i.e. 5.5%, had anti-hepatitis B titre less than 10, hence, were advised HBV immunoglobulin, and the vaccination schedule was repeated.

Among the partially vaccinated healthcare workers (38.4%), 90% had protective titre and were advised to complete the vaccination schedule. The remaining 10% had a less than 10 titre level. Therefore, they were given HBV immunoglobulin and were advised to complete the vaccination schedule. All unvaccinated were given HBV immunoglobulin and were advised to take hepatitis B vaccination.

HIV

Among the possible HIV-positive exposures (16+54 = 70), all the healthcare workers were advised to take post-exposure prophylaxis. Two persons were not willing, and the rest (97.1%) initiated PEP. Among this group, 66 (97%) completed PEP, and the rest did not complete it. Thankfully, none of them showed seroconversion till the sixth month's follow-up.

Hepatitis C

Among the HCWs with possible HCV-positive exposures (14+54 = 68), all the HCWs were given counseling and advised to follow up. No seroconversion at six months of follow-up has been recorded till now.

## Discussion

A total of 189 healthcare workers reported exposures in the last five years. Various studies have reported a different total number of exposures, like 476 reported instances over three years as reported by Goel et al. [8], and 380 instances in six years as reported by Mehta et al. [9]. As the nature of services varies in each hospital, no significance can be drawn by comparing numbers from different settings.

Although the majority of the healthcare workers who reported were female (84.1%), this is in line with the fact that more than 80% of healthcare workers in the hospital are females at any given point in time.

Among the healthcare workers who reported exposure to blood and body fluids, the majority of them, i.e. 44.4%, were from the nursing department. This is consistent with the findings of many other studies by Mehta et al. [9], Sharma et al. [6], and Singru and Banerjee [10] where nurses were the most common HCW exposed to splash or injury, constituting 45%, 61.5%, and 39.5%, respectively. This might be due to the nurses contributing a larger proportion of the health workforce and their constant and regular presence during most procedures, including injections, dressings, invasive procedures, handling IV lines, etc. The second most common group was housekeeping staff.

The most common exposure we found was needle prick (86.2%), followed by splash of fluids. Thus, injury by needle was the most common type of exposure, and almost all studies agree with this finding. However, there is variation when it comes to the second most common type. Splash of fluid was reported as the second most common type in some studies [6, 10, 11], while others reported sharp injury as the second most common [8, 12]. This variation might be explained by the change in exposure with differences in the type of services provided in each center and the type of waste generated as a consequence.

The majority of exposures, i.e. 26.4%, were during the transfer of sharps or while cleaning, which was almost exclusively reported by housekeeping staff. If housekeepers were excluded, then the most common instance of exposure was drawing blood, followed by minor/major procedures. Blood withdrawal was reported as the most common activity associated with injury by Muralidhar et al. [13] and Makade et al. [14]. Injury during procedures was reported as the majority by Akthar et al. [15]. Handling of needles was found to be the major factor leading to injury in studies done by Sharma et al. [16] although only 15% was found in our study. Similarly recapping, which is reported by only 15%, was found to be the major factor by Shriyan and Annamma [11]. Other major contributors were intravenous line insertion, as reported by Mehta et al. [9], and administering parenteral medication, as reported by Sharma et al. [6] although they were not in the majority in our study.

We found that the most commonly reported place of exposure was the Emergency Department and Intensive Care Units (ICU) (30.3%), followed by inpatient wards. Sharma et al. [6] reported a similar higher proportion (41%) of cases in critical units and emergency rooms in a multi-specialty tertiary care centre. Other studies report wards as the place where the majority of exposure happens [7, 12, 15, 17]. Emergency and ICUs are locations where speed of work is an essential part of care. Consequently, there is more chance of accidents in these locations.

Among the study subjects, 66% were immunized for hepatitis B, and this was similar to findings by Makade et al. [14] who found 68.9% to be immunized, and Singru and Banerjee [10] who found 54% to be vaccinated. We have instituted a policy of compulsory vaccination against hepatitis B for all new employees and exemption to vaccination will be provided only for those with a protection level of Anti-Hep B titres.

The serological status of the source was known in 71.4% of reported exposures in our study, and similar results were reported by Mehta et al., i.e. 67% [9], while Shriyan and Annamma [11] found a lower level of 52.5%.

A total of 99.5% of healthcare workers washed the wound immediately after the exposure. This was far better than reported by Sriram [12] and Sharma et al. [16] who reported that 82% and 60.9% took immediate action after exposure, respectively. This might suggest a higher level of knowledge regarding immediate action among our study subjects.

Among the post-exposure reports in our study, where the serological status of the source was known, hepatitis B was the most common (17.8%), followed by HIV. Mehta et al. [9] and Goel et al. [8] reported a similar pattern of hepatitis B followed by HIV. However, Shriyan and Annamma [11] reported a significant higher proportion of HIV (22.6%) compared to hepatitis B (9.6%).

Among the HIV-positive exposure from known source, more than 97.1% of healthcare workers initiated post-exposure prophylaxis (PEP). This was in contrast to the finding by Singru and Banerjee [10] who found that only 50% of HCWs were started on PEP when indicated. Among those who started PEP, 97% completed the course and were found to be negative, a significantly higher proportion compared to 73.1% reported by Sin et al. [18]. This also shows the effect of actions taken by infection control personnel in instilling the importance of proper action after exposure to the HCWs.

Limitations

Our study is a record-based study, and hence information like the incidence of injury among healthcare workers, the non-reporting rate following exposure, etc., is outside the scope of this study. As with all record-based studies, the quality of data will vary based on the interviewer, though the use of a standard questionnaire reduces inconsistency. Also, since the data was collected immediately after the exposure, recall bias is reduced. The factors leading to exposure seem to vary from centre to centre and hence the findings of our study are not generalizable.

## Conclusions

Healthcare workers are constantly at risk of exposure to splash, sharp and needle-stick injuries, and although all categories of workers are at risk, nurses are at particularly high risk. A variety of activities can result in injury or a splash of fluids and so preventive activities, including health education, should be focused on all areas of healthcare and for all healthcare workers. The nature of the work seems to determine the type of activity associated with injury or exposure. Hence a one size fits all model cannot be followed in health education.

Although the proportion of healthcare workers who took proper post-exposure prophylactic measures is higher in our study compared to others, the aim is to achieve full coverage. As these measures are voluntary, more awareness is needed among healthcare workers regarding post-exposure prophylaxis. Although it is outside the scope of our study, it’s seen from other studies, that healthcare workers are not keen on reporting exposure and thus, do not receive the required care. Thus, continuous and specific health educations of healthcare workers become even more important.
